# Patellar resurfacing is associated with reduced postoperative effusion compared with synovectomy in severe chondrocalcinosis undergoing total knee arthroplasty

**DOI:** 10.1007/s00264-026-06767-6

**Published:** 2026-03-03

**Authors:** Philippe Hernigou, Nizar Touati, Charles-Henri Flouzat-Lachaniette

**Affiliations:** Paris-Est Créteil University, Paris, France

**Keywords:** Patella resurfacing, Chondrocalcinosis, Total knee arthroplasty, Joint effusion, Synovectomy

## Abstract

**Purpose:**

Regarding patellar resurfacing in total knee arthroplasty (TKA), no consensus has been reached, but most studies have not addressed specific pathological circumstances. Evidence on the roles of patellar resurfacing and synovectomy in managing postoperative effusion in patients with severe chondrocalcinosis is limited.

**Material and methods:**

This single-centre observational cohort study included 160 patients who underwent the same TKA for osteoarthritis with severe chondrocalcinosis (grade 4) between January 2000 and December 2010. A matched design created four comparable groups of 40 patients each: (1) TKA without patellar resurfacing or synovectomy, (2) TKA with patellar resurfacing alone, (3) TKA with synovectomy alone, and (4) TKA with both patellar resurfacing and synovectomy. Severe chondrocalcinosis (advanced calcium pyrophosphate deposition disease) was confirmed through radiographic findings, synovial fluid analysis using polarized light microscopy, and histology. Significant postoperative effusion was diagnosed with ultrasound, quantified by sterile joint aspiration, and classified as stage I (10–20 cm^3^), stage II (21–30 cm^3^), or stage III (> 30 cm^3^).

**Results:**

Postoperative joint effusion varied significantly between the strategies. In the patellar resurfacing group, 25% (10/40) of patients developed only stage I effusion without synovectomy. Conversely, 45% (18/40) of patients in the synovectomy-only group developed stage II effusion, while 62.5% (25/40) of patients without either procedure developed stage III effusion (*p* < 0.0001). TKA with both patella resurfacing and synovectomy resulted in either stage I (7/40) or stage II effusion (6/40). Multivariate regression confirmed patellar resurfacing as an independent protective factor against postoperative effusion (*p* < 0.01). Average aspirated effusion volumes further supported these findings: 39 ± 6 cm^3^ for TKA without additional procedures, 18 ± 8 cm^3^ with synovectomy, 6 ± 4 cm^3^ with patellar resurfacing, and 7 ± 4 cm^3^ with both patellar resurfacing and synovectomy. The results showed that as total knee effusion volume increased, inflammatory markers (C-reactive protein level) increased, and range of motion decreased.

**Conclusion:**

In severe chondrocalcinosis, patellar resurfacing may be appropriate to prevent joint effusion after TKA.

## Introduction

From a clinical perspective, persistent joint effusion after TKA is not a benign condition [1]. It often results in repeated visits, infection assessments, and increased patient anxiety. When significant fluid accumulation occurs, aspiration may be necessary for symptom relief and diagnosis. Finally, detecting a large effusion can increase patient anxiety, as it raises concerns about complications and the possible need for additional treatment.

Among the multiple causes of joint effusion after TKA, chondrocalcinosis **(**CPPD), despite being quite common [2, 3] in osteoarthritis (around 30% of patients), remains underexplored. While TKA effectively restores function and relieves pain, postoperative inflammatory reactions—especially joint effusion—are still frequently observed in patients with chondrocalcinosis [4–6]. Surgical strategies to reduce this response have included synovectomy [7], aimed at lowering the synovial inflammatory load, but it is now typically discussed only in cases of severe chondrocalcinosis [8].

When patella resurfacing is not performed, persistent effusion after TKA in knees with chondrocalcinosis could also occur due to retained crystal deposits in the patellar cartilage. Although many studies discuss the pros and cons of patellar resurfacing [9, 10], none have specifically examined its effect—compared to synovectomy—on postoperative joint effusion in patients with advanced calcium pyrophosphate deposition disease (CPPD).

This study aims to 1) evaluate effusion in patients with severe chondrocalcinosis; 2) examine the effect of four different surgical strategies on the amount of joint effusion obtained through joint aspiration in patients with severe chondrocalcinosis: TKA without patellar resurfacing or synovectomy, TKA with patellar resurfacing alone, TKA with synovectomy alone, and TKA with both patellar resurfacing and synovectomy; and 3) assess the overall impact of these surgical strategies on complications and implant survivorship.

## Materials and methods

This retrospective, single-center observational cohort study included 160 patients who underwent primary total knee arthroplasty (TKA) for osteoarthritis with severe chondrocalcinosis (grade 4) between January 2000 and December 2010. These 160 patients were selected from a database of 1800 total knee arthroplasties (TKAs) performed by experienced surgeons.

### Materials

#### Chondrocalcinosis diagnosis

**Fig. 1:**
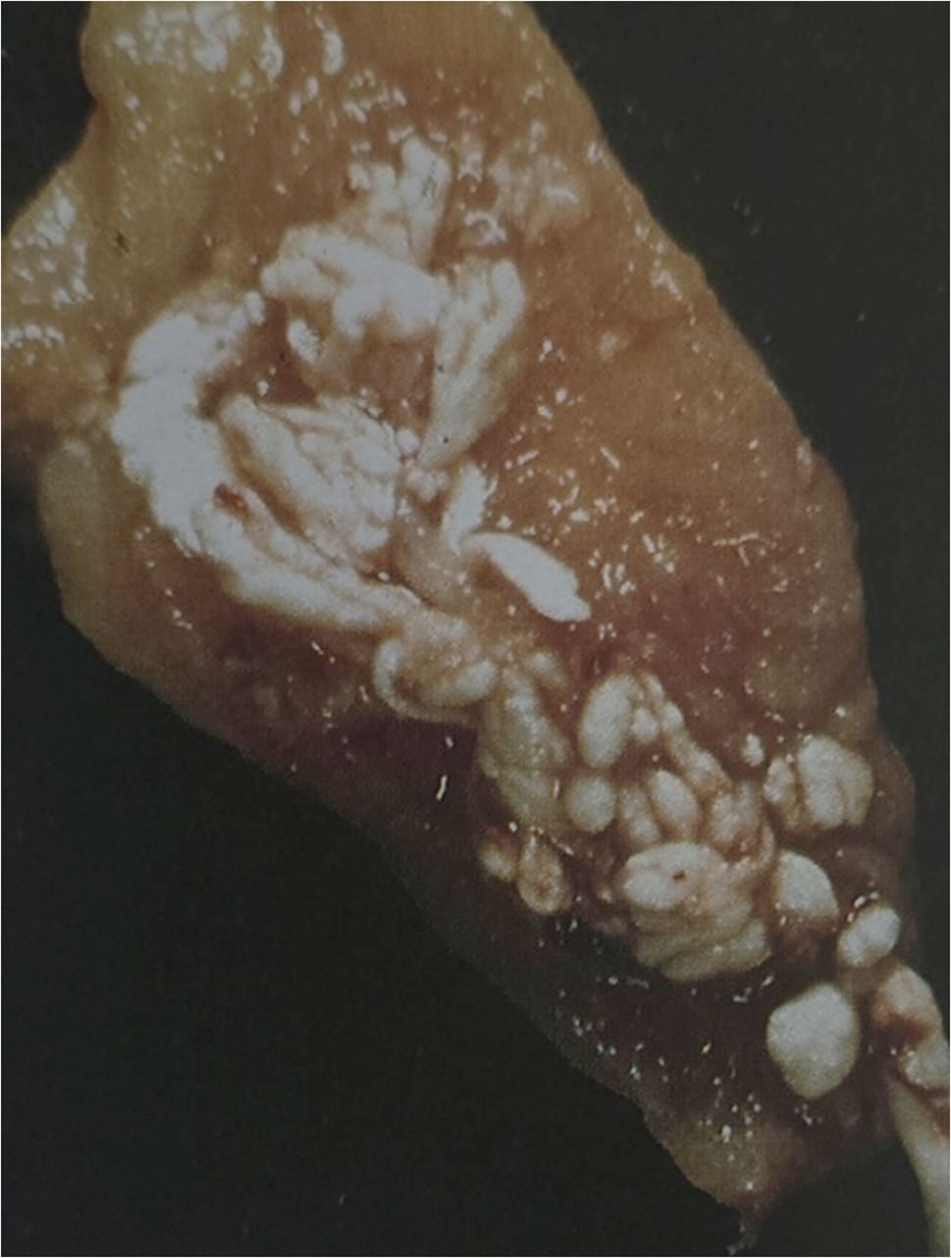
Synovial tissue: Example of severe chondrocalcinosis (grade 4)

Chondrocalcinosis was suspected based on radiographic findings. Confirmation was obtained through synovial fluid analysis with polarized light microscopy, histology, intraoperative assessment, standard radiographs, and analysis of the synovial tissue, showing visible CPPD crystal deposits [11–13]. The severity of chondrocalcinosis was graded on a visual scale based on the amount of visible crystal deposits in the synovium at the time of surgery, ranging from 0 to 4: 0 = none, 1 = minimal, 2 = mild, 3 = moderate, and 4 = severe. The grading was based on the number of calcific deposits in the synovium. When at least five calcific deposits of 5 mm in diameter were visible during surgery Fig. 1 or on the synovial tissue, knees were classified as severe (grade 4) and included in the analysis.

#### Study design

The indications for TKA were osteoarthritis. The study excluded patients with rheumatoid arthritis, pre- or postoperative infections, and revision surgeries. Because there are no guidelines or expert consensus on patella resurfacing for patients undergoing TKA and synovectomy with severe chondrocalcinosis, our centre has no specific criteria for either procedure in such cases. Consequently, doctors decided their surgical strategy based on their clinical experience.

**Fig. 2:**
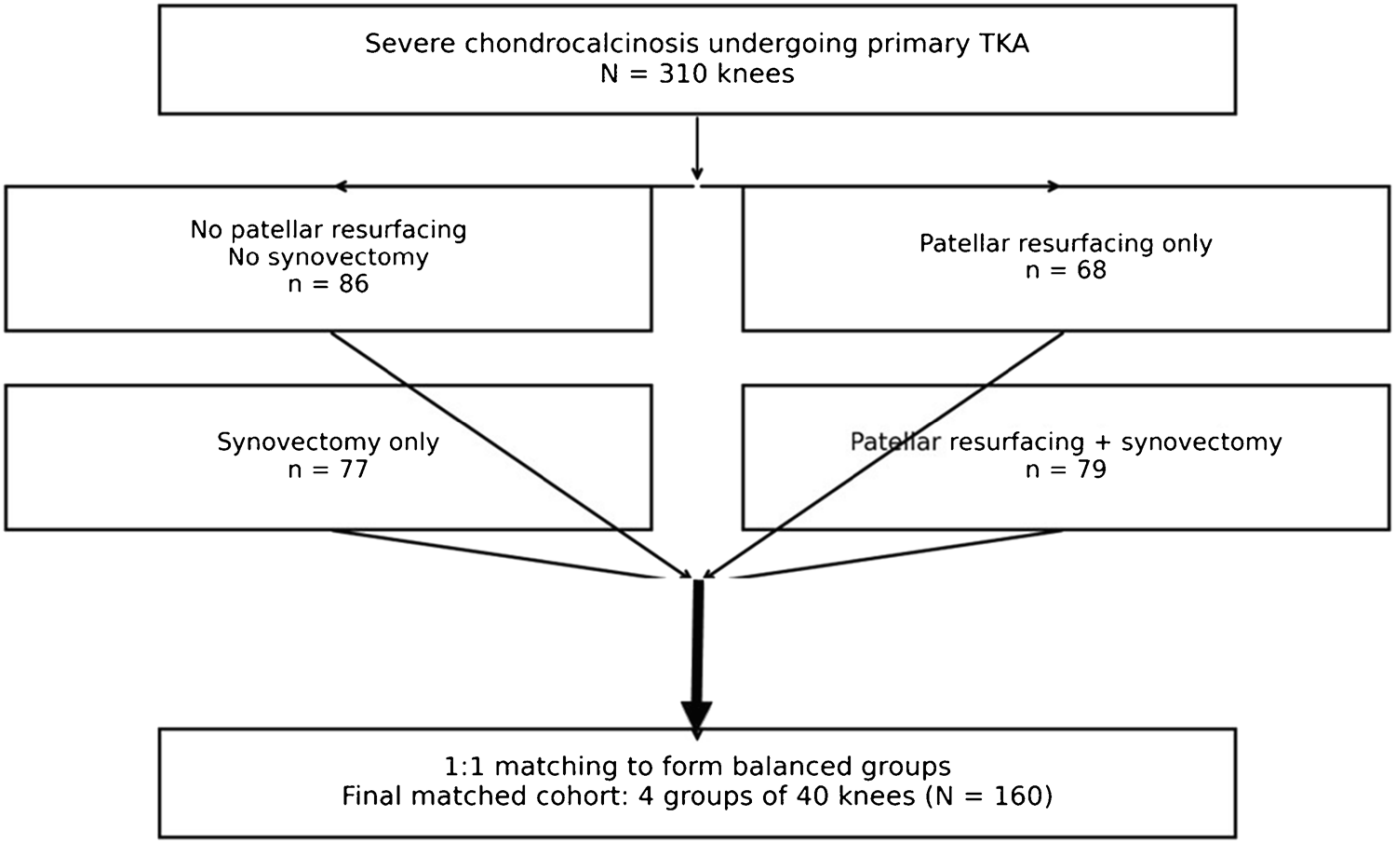
Study flow: Among 310 knees with severe chondrocalcinosis undergoing primary total knee arthroplasty (TKA), four surgical strategies were identified: no patellar resurfacing (PR) and no synovectomy (*n* = 86), PR only (*n* = 68), synovectomy only (*n* = 77), and combined PR plus synovectomy (*n* = 79)

Out of 1800 knees in the database, 824 had chondrocalcinosis, and 310 had severe chondrocalcinosis. Among the 310 knees with severe chondrocalcinosis Fig. 2, 86 received TKA without patellar resurfacing or synovectomy, 68 had TKA with patellar resurfacing alone, 77 had TKA with synovectomy alone, and 79 had TKA with both patellar resurfacing and synovectomy. To evaluate joint effusion, we selected patients who underwent a single TKA, had no evident complications or revisions. Anti-inflammatory drugs, corticosteroids, and colchicine, if any, were discontinued 2 weeks before evaluating the joint effusion.

A matched design was used to create four comparable groups of 40 patients each: (1) TKA without patellar resurfacing or synovectomy, (2) TKA with patellar resurfacing alone, (3) TKA with synovectomy alone, and (4) TKA with both patellar resurfacing and synovectomy.

Because patellar resurfacing and synovectomy were not assigned randomly, a propensity score–based matching method was employed to reduce confounding related to treatment decisions. Propensity scores were derived using a multinomial logistic regression model that included baseline variables considered clinically relevant to postoperative effusion and outcomes, such as age, sex, body mass index, osteoarthritis phenotype, severity of patellofemoral osteoarthritis with Kellgren and Lawrence (KL grade) classification [14], postoperative limb alignment with hip knee ankle (HKA) angle, and the same implant size. Patients were matched to create four balanced treatment groups corresponding to the four surgical strategies Fig. 2: no patellar resurfacing and no synovectomy; patellar resurfacing alone; synovectomy alone; and combined patellar resurfacing with synovectomy. Covariate balance after matching was assessed using standardized mean differences (SMDs), with values below 0.10 indicating acceptable balance.

#### Patients’ demographics

Among the 310 knees with severe chondrocalcinosis,

Fifty-five patients underwent bilateral knee replacements. The average age at surgery was 75 years (range, 65–90 years). The mean follow-up period was 16 years, ranging from 13 to 24 years. To ensure an accurate estimate of effusion prevalence in this population, no patient in this series was excluded from the final analysis. Patients were followed at regularly scheduled intervals, and clinical data were prospectively collected.

For knees with synovectomy, during knee exposure, the synovium in the suprapatellar pouch and the medial and lateral gutters was sharply excised and separated from the joint capsule. After completing the bony resections, the posterior knee synovium was removed with a rongeur.

All procedures used the same implant design and manufacturer (Ceraver Osteal, Roissy, France), and followed a standardized surgical technique for cementing all implants. All the implants were poster-stabilized and were manufactured by the same company.

*In the four treatment groups (160 knees)*, baseline demographic and preoperative characteristics were comparable across groups Table 1.

**Table 1:** Baseline demographic and clinical characteristics of the four surgical groups

Variable	No PR/No Syn (*n* = 40)	PR Only (*n* = 40)	Syn Only (*n* = 40)	PR + Syn (*n* = 40)	Standardized Mean Difference (max)
Age (years), M ± SD	73.8 ± 6.1	74.2 ± 5.8	74.1 ± 6.0	74.0 ± 6.2	< 0.10
Sex (female), n (%)	27 (67.5%)	29 (72.5%)	28 (70.0%)	28 (70.0%)	< 0.10
BMI	27.8 ± 3.9	28.1 ± 4.1	28.0 ± 4.0	28.2 ± 3.8	< 0.10
OA phenotype					
– Genu varum, n (%)	35 (87.5%)	37 (92.5%)	36 (90.0%)	36 (90.0%)	< 0.10
– Genu valgum, n (%)	5 (12.5%)	3 (7.5%)	4 (10.0%)	4 (10.0%)	< 0.10
Patellofemoral OA					
– Grade 1, n (%)	11 (27.5%)	9 (22.5%)	10 (25.0%)	10 (25.0%)	< 0.10
– Grade 2, n (%)	22 (55.0%)	25 (62.5%)	24 (60.0%)	23 (57.5%)	< 0.10
– Grade 3, n (%)	7 (17.5%)	6 (15.0%)	6 (15.0%)	7 (17.5%)	< 0.10
Alignment	174.8 ± 4.2	175.2 ± 3.9	175.0 ± 4.0	175.1 ± 4.1	< 0.10

*PR* patellar resurfacing, *Syn* synovectomy, *OA* osteoarthritis (KL grade). Alignment (degrees), mean ± SD. *BMI* Body Mass Index (kg/m^2^), mean ± SD. Covariate balance was assessed using standardized mean differences (SMD), with values < 0.10 indicating adequate balance across groups.

### Methods of evaluation of the different strategies' outcomes

In the absence of published data defining the expected volume of joint effusion several years after total knee arthroplasty, we conducted a prospective preliminary assessment in six patients at ten years after TKA. Inclusion criteria were: absence of chondrocalcinosis on radiographs of the contralateral knee, a pain-free prosthesis, no clinical signs of infection or instability, and normal inflammatory markers, C-reactive protein (CRP) levels. Three knees were with patella resurfacing, and three knees without patella resurfacing.

Effusion volume was first estimated using standardized ultrasound measurements of the suprapatellar recess. Subsequently, diagnostic joint aspiration was performed under sterile conditions, and the aspirated volume was recorded. The aspirated volumes were used to calibrate and validate the ultrasound-based measurements. These values were considered to represent physiological postoperative effusion in asymptomatic total knee arthroplasty: with patella resurfacing, 5 cc, 10 cc, 15 cc; without resurfacing, 4 cc, 11 cc, 14 cc. The volume was graded as low on ultrasound in all cases.

#### First outcome: Joint effusion evaluation on the selected 160 knees

Among the selected knees, joint effusion stability was assessed with a question at approximately ten years of follow-up, and each joint underwent a second clinical evaluation at one month intervals. The Knee Society Score (KSS) was recorded at the first visit.

At the second visit, the KSS was reassessed. If the score was identical to that obtained at the first evaluation, the volume of joint effusion was measured by ultrasound prior to aspiration. During the same visit, C-reactive protein (CRP) levels were obtained.

Joint effusion was evaluated through a standardized ultrasound exam [15], with the patient lying on their back and the knee slightly flexed at 20–30°. A high-frequency linear transducer was positioned lengthwise over the suprapatellar pouch. Care was taken not to press too hard with the probe to prevent underestimating the intra-articular fluid. Joint effusion was defined as the presence of compressible, clear (anechoic), or dark (hypoechoic) intra-articular material without a Doppler signal and was graded as low, significant, or high volume effusion by the examiner.

After joint aspiration, the fluid was analyzed for microcrystalline arthropathy [11–13], haemarthrosis, infection, and wear debris (small polyethylene particles) that can irritate the synovium (debris-induced synovitis), as well as other causes such as metallosis. The severity of postoperative joint effusion was classified as stage I (< 10 cm^3^), stage II (10–30 cm^3^), or stage III (> 30 cm^3^).

The correlation between ultrasound and aspiration volume, and the effects of effusion on CRP and range of motion, were assessed.

#### Second outcome: Complications and survivorship among the 310 knees with severe chondrocalcinosis

Postoperative complications were systematically recorded and compared across the four different strategies, including the 310 knees before matching, for both complications and survivorship. Among the 310 knees with severe chondrocalcinosis Fig. 2, 86 received TKA without patellar resurfacing or synovectomy, 68 had TKA with patellar resurfacing alone, 77 had TKA with synovectomy alone, and 79 had TKA with both patellar resurfacing and synovectomy.

Deep prosthetic infection was defined as positive cultures from synovial fluid or periprosthetic tissue samples, or treatment initiated based on clinical presentation, such as antibiotics, aspiration, lavage, polyethylene insert exchange, or prosthesis removal.

Revision was defined as the partial or complete removal of the prosthesis, with implantation of a new component or a total prosthesis. Reoperation was defined as any surgical procedure performed on a knee with the prosthesis in situ that did not involve component removal or exchange.

In cases of reduced postoperative range of motion, manipulation under anesthesia was performed.

### Statistical analysis

Continuous variables are shown as mean values with ranges or standard deviations (SD), or medians with interquartile ranges (IQR), depending on data distribution. Categorical variables are presented as counts and percentages. Normality was evaluated through visual inspection of histograms and the Shapiro–Wilk test.

To assess the relationships between surgical strategy and effusion severity, multivariable regression analyses were conducted within the matched cohort. The primary analysis employed ordinal logistic regression, treating effusion stage as an ordered categorical outcome. Patellar resurfacing and synovectomy were included as main effect variables. Results are presented as odds ratios (ORs) with 95% confidence intervals (CIs), indicating the likelihood of experiencing a higher severity stage of effusion. As a complementary confirmatory analysis, effusion severity was dichotomized into mild (stage I) versus clinically significant effusion (stage II–III), and multivariable logistic regression was performed using the same covariates. This binary approach was chosen to reflect clinically meaningful thresholds commonly prompting diagnostic or therapeutic interventions. Postoperative effusion volume, measured as the amount of aspirated fluid in cubic centimeters, was analyzed as a continuous variable. Between-group comparisons were conducted using one-way analysis of variance (ANOVA) or the Kruskal–Wallis test when appropriate, followed by post hoc;

Simple linear regression analyses were conducted to assess potential correlations between knee effusion volume and inflammatory markers, as well as between knee effusion volume and joint function.

Implant survival was analyzed using Kaplan–Meier methods, with revision or any reoperation as endpoints, and survival curves were compared using the log-rank test. Cox proportional hazards regression was performed in exploratory analyses to assess associations between surgical strategy and implant survival. Functional outcomes were compared using appropriate parametric or non-parametric tests according to data distribution.

All statistical tests were two-sided, and a p value < 0.05 was considered statistically significant.

## Results

Microcrystalline Arthropathy (aspirated CPPD-positive knees) was confirmed by synovial fluid analysis with polarized light microscopy [11–13] for all 160 knees. After secondary biological analysis, another possible cause of effusion was identified in 20 knees: haemarthrosis in six knees; 12 knees with wear debris, and two with metallosis.

### General outcomes on effusion measurement in severe chondrocalcinosis

All knees had some effusion, always detectable by clinical exam, ultrasound, and joint aspiration. Microcrystalline Arthropathy (aspirated CPPD-positive knees) was confirmed by synovial fluid analysis with polarized light microscopy [11–13] for all 160 knees. After secondary biological analysis, another possible cause of effusion was identified in 20 knees: haemarthrosis in six, wear debris in 12, and metallosis in two. These other causes were considered minor and excluded from the analysis.

#### Ultrasound imaging and joint aspiration volume

The persons who performed the ultrasound exam graded the effusion as low in 40 knees, significant in 50, and high volume in 70 knees. The corresponding volume of joint effusion obtained by aspiration were average 12 cc (range 4 to 15 cc) for low effusion, 25 cc (range 12 to 35 cc) for significant effusion and 34 cc (range 24 to 62cc).

**Fig. 3:**
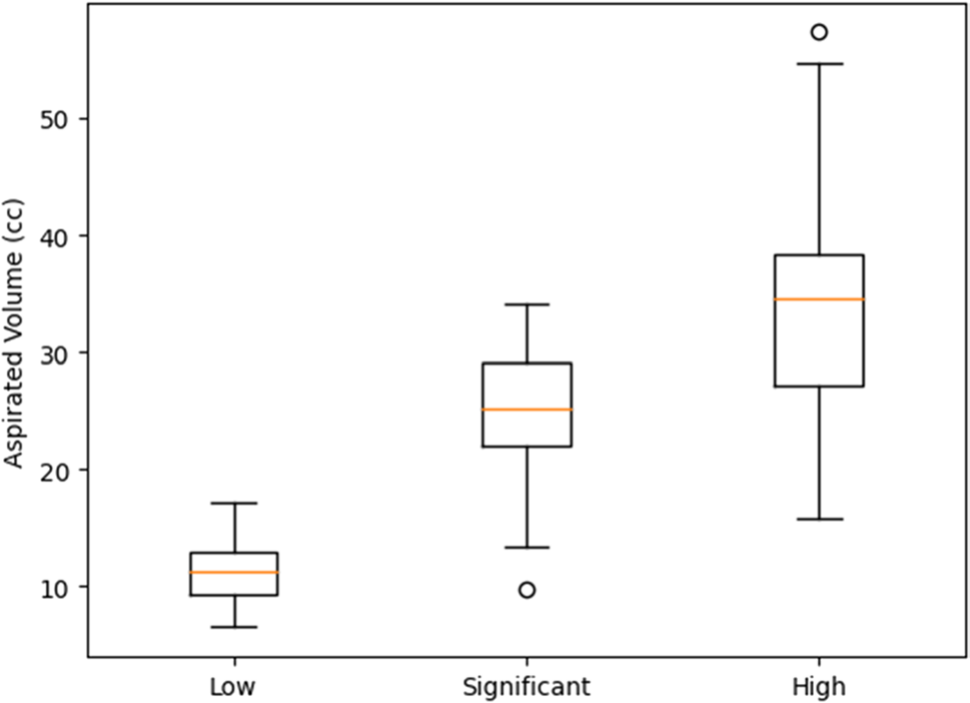
Boxplot representation of aspirated joint volumes according to ultrasound effusion grade on the X axis, and volume of aspiration on the Y axis. Limited overlap was observed between adjacent categories, whereas none occurred between the low and high groups

Ultrasound grading showed Fig. 3 a clear stepwise association with aspirated joint volume. Mean aspirated volumes increased progressively from 12 cc (range 4–15 cc) in the low-effusion group to 25 cc (12–35 cc) in the significant-effusion group and 34 cc (24–62 cc) in the high-volume group. No overlap was observed between low and high effusion categories, while limited overlap occurred between adjacent groups. These findings support a strong ordinal relationship between ultrasound assessment and true intra-articular fluid volume, suggesting good discriminative validity of the ultrasound grading system. A one-way ANOVA demonstrated a highly significant difference in aspirated joint volume across ultrasound grades (*p* < 0.001).

#### Relationship between knee effusion and CRP or ESR value

**Fig. 4:**
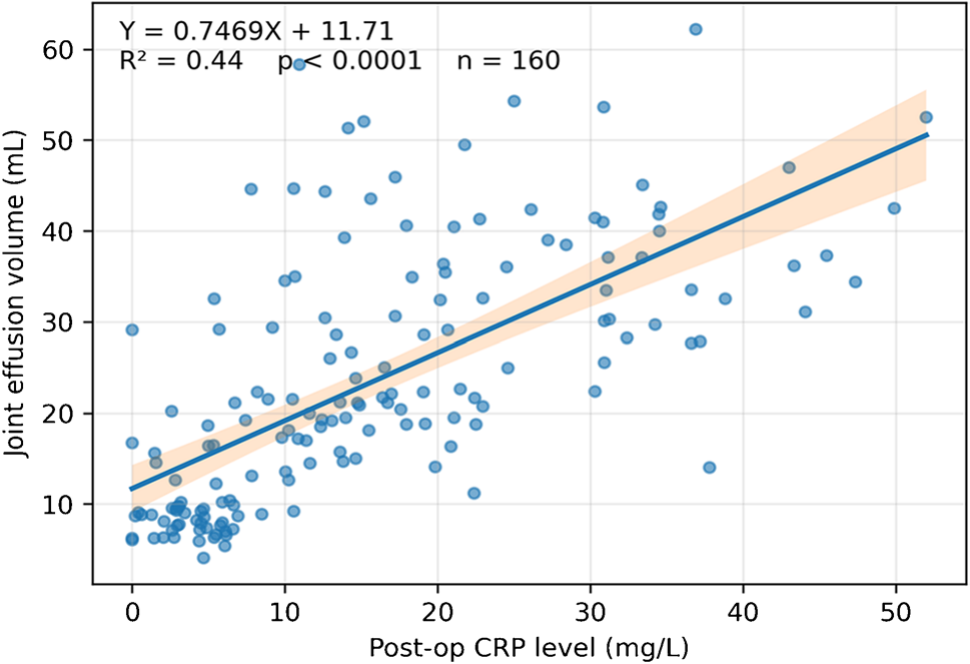
Positive correlations were observed between CRP levels and knee effusion volume

The mean serum inflammatory marker levels of the patients were significantly elevated (CRP, 35.5 ± 12.3 mg/L, range 1 to 53). The results showed that as total knee effusion volume increased, CRP levels increased (Fig. 4).

#### Relationship between knee effusion and range of motion (ROM)

**Fig. 5:**
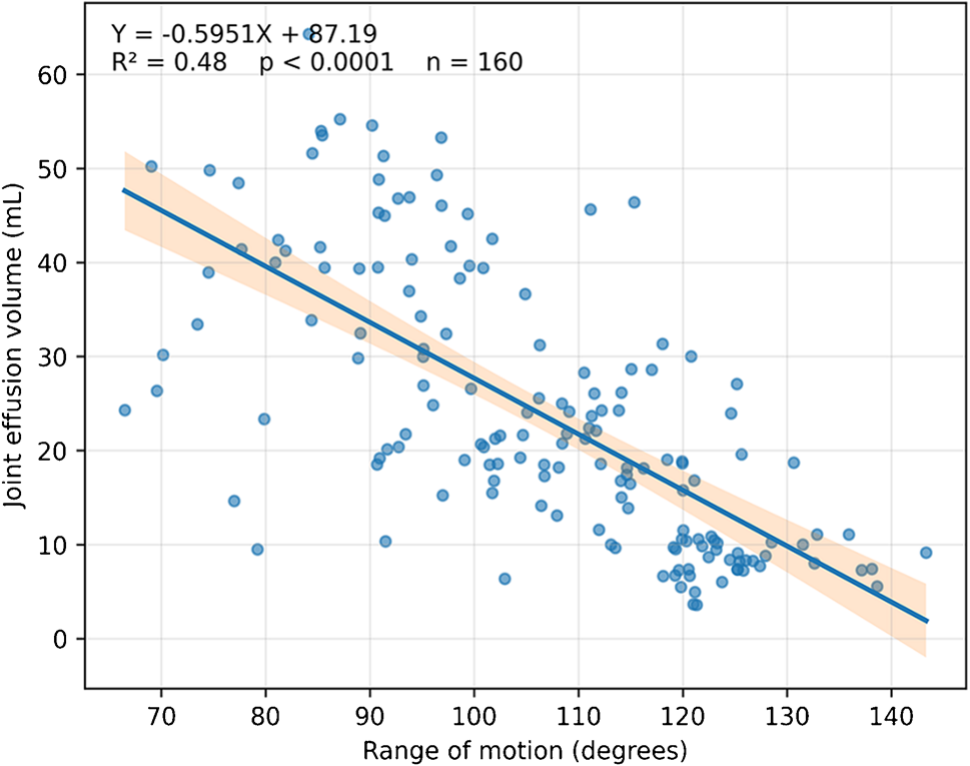
A negative correlation was found between knee effusion volume and the range of motion

The mean knee ROM was 116.1° ± 15.46°, range 65° to 145°. Using joint knee effusion volume at 10 years follow-up, a negative correlation Fig. 5 was observed between the knee effusion volume and the actual value of the ROM (*p* = 0.0001).

### Surgical strategy and joint effusion (160 knees)

**Fig. 6:**
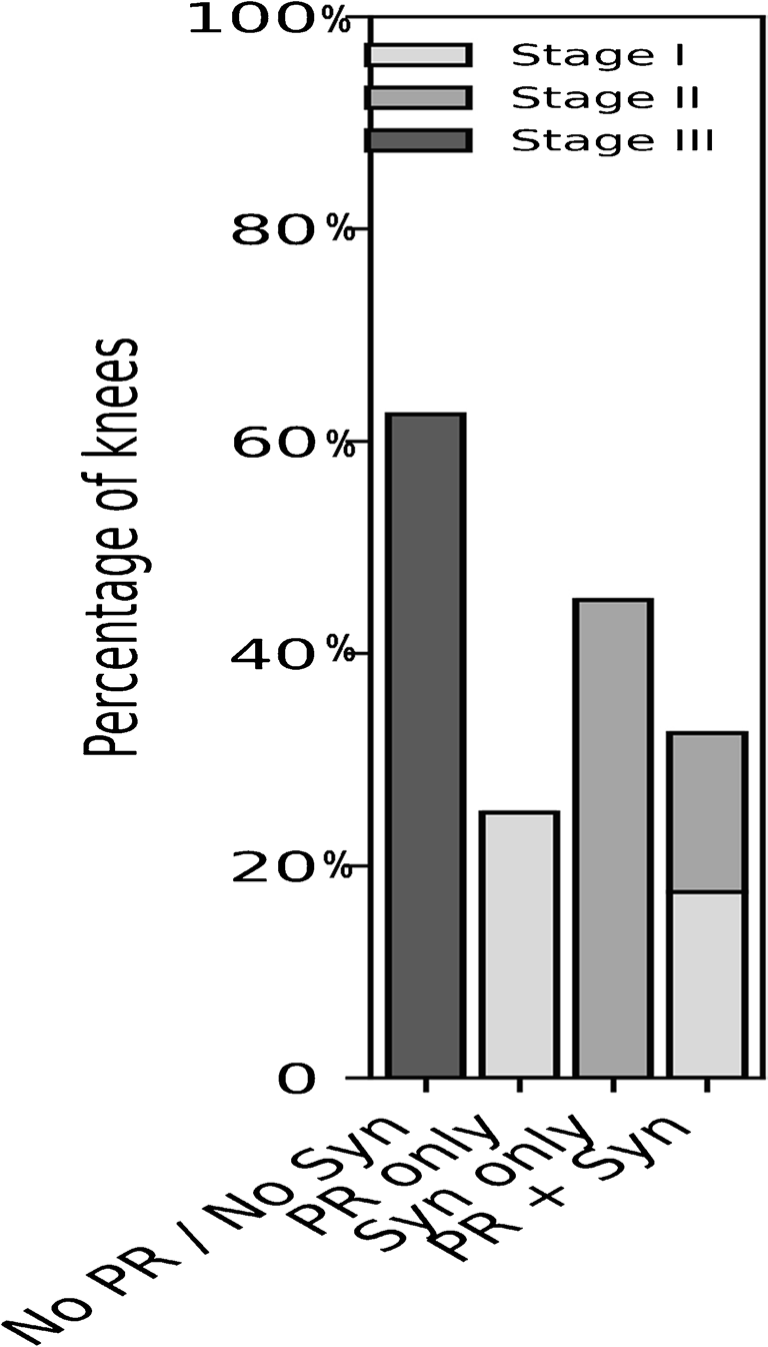
Postoperative joint effusion frequency; PR = patella resurfacing

Effusion Fig. 6 varied significantly depending on the surgical approach (*p* < 0.01). In knees treated without patellar resurfacing or synovectomy, 62.5% (25/40) developed stage III effusion (> 30 cm^3^). In the synovectomy-only group, 45% (18/40) experienced stage II effusion (21–30 cm^3^). The patellar resurfacing–only group mainly had mild effusion, with 25% (10/40) limited to stage I (10–20 cm^3^).

Knees treated with both patellar resurfacing and synovectomy mostly showed effusion restricted to stage I (7/40) or stage II (6/40), with no cases of stage III effusion.

**Fig. 7:**
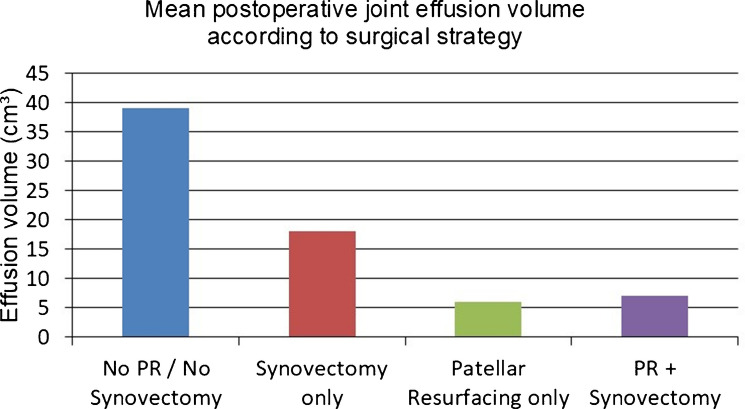
Average postoperative joint effusion volume based on surgical strategy in patients with severe chondrocalcinosis. Patellar resurfacing (PR), with or without synovectomy, resulted in a significant reduction in effusion volume compared to synovectomy alone or no additional procedure

Mean aspirated effusion volumes mirrored these findings Fig. 7:

- 49 ± 6 cm^3^ in the knees without additional procedures
- 28 ± 8 cm^3^ with synovectomy alone
- 16 ± 4 cm^3^ with patellar resurfacing alone
- 17 ± 4 cm^3^ with both patellar resurfacing and synovectomy

Multivariate regression analysis confirmed that patellar resurfacing was an independent protective factor against clinically significant postoperative effusion (*p* < 0.01). Synovectomy showed a more modest and inconsistent association with reduced effusion and did not eliminate advanced effusion. In multivariate logistic regression, patellar resurfacing was independently associated with a lower risk of stage II–III effusion (OR 0.28; 95% CI 0.12–0.64; *p* = 0.003).

### Overall effect of surgical strategy on complications and implant survivorship (310 Knees)

To evaluate the broader clinical safety of each surgical strategy, complications and implant survivorship were analyzed in the entire cohort of 310 knees with severe chondrocalcinosis prior to matching. This approach allowed assessment of long-term implant durability and adverse events without the reduction in sample size inherent to propensity matching. Among the 310 knees, 86 underwent TKA without patellar resurfacing (PR) or synovectomy, 68 had PR alone, 77 underwent synovectomy alone, and 79 received combined PR and synovectomy.

*Implant Survivorship:* At 10 years, cumulative implant survival with revision as the endpoint was:

- 97.4% (95% CI, 96.8–99.0) in knees without PR or synovectomy
- 96.0% (95% CI, 95.1–97.9) in knees with PR alone
- 88.0% (95% CI, 84.9–92.1) in knees with synovectomy alone
- 91.4% (95% CI, 88.6–93.1) in knees treated with both PR and synovectomy

Although numerical differences in survivorship were observed, particularly lower survival in synovectomy-only knees, confidence intervals overlapped between groups. Kaplan–Meier analysis demonstrated no statistically significant difference in ten year implant survivorship (log-rank test, *p* > 0.05).

Overall, severe chondrocalcinosis did not compromise long-term implant survival when modern cemented posterior-stabilized implants were used.

#### Reoperations and complications

When reoperations (including procedures not involving component exchange) were considered as endpoints, survival was significantly lower in knees treated with synovectomy (*p* = 0.01).

In the synovectomy-only group, complications requiring reintervention without implant removal included: five cases of stiffness requiring manipulation under anaesthesia, two deep infections, four severe postoperative haematomas, six cases of patella baja. These findings suggest increased soft-tissue morbidity associated with extensive synovial resection.

Patella-related reoperations were limited. In the PR group, two procedures were performed for patellar instability. In knees without primary PR, six patients later required secondary patellar resurfacing for anterior knee pain.

#### Functional outcomes

At final follow-up, knees treated without synovectomy demonstrated superior functional outcomes. The Knee Society Knee Score was significantly higher in non-synovectomy knees compared with synovectomy knees (89.1 vs 81.3 points; *p* = 0.02), and flexion range of motion was greater (129° vs 105°; *p* = 0.01).

These findings indicate that while synovectomy did not improve effusion control or implant survival, it was associated with higher complication rates and inferior functional recovery.

### Integrated interpretation

To further explore the independent association between surgical strategy and implant failure, a Cox proportional hazards regression analysis was performed using revision as the endpoint. The model included patellar resurfacing (yes/no), synovectomy (yes/no), age, sex, body mass index, limb alignment (HKA angle), and severity of patellofemoral osteoarthritis as covariates.

In unadjusted analysis, synovectomy was associated with an increased hazard of revision (HR 1.82; 95% CI 1.01–3.29; *p* = 0.046), whereas patellar resurfacing was not associated with a higher risk of revision (HR 0.94; 95% CI 0.48–1.84; *p* = 0.86).

Taken together, these results demonstrate three important points:

- Patellar resurfacing did not compromise implant survival and was not associated with increased complication rates.
- Synovectomy was associated with higher morbidity and lower reoperation-free survival, without measurable benefit in long-term implant durability.
- Severe chondrocalcinosis itself did not reduce 10-year implant survivorship, reinforcing that TKA remains a reliable procedure in this population when modern techniques and implants are used.

These data support the concept that, in severe CPPD, controlling the biological source of crystal shedding (patellar cartilage) may be more relevant than extensive synovial excision for optimizing outcomes.

## Discussion

Chondrocalcinosis, most often associated with calcium pyrophosphate dihydrate (CPPD) crystal deposits, is commonly seen in patients undergoing total knee arthroplasty (TKA) for osteoarthritis. The earliest reports on CPPD date back to the 1960 s [16], yet only a few studies (Table 2) have examined outcomes related to this condition after arthroplasty [17–25]. The main findings of this study are that effusion is frequent in TKA with chondrocalcinosis and that the surgical approach significantly affects postoperative joint effusion after total knee arthroplasty (TKA) in patients with severe chondrocalcinosis.

**Table 2:** Studies with arthroplasty mentioning chondrocalcinosis

**Variable**	Weeds (1995)	Pandit (2011)	Hernigou (2012)	Pandit (2016)	Hamilton (2017)	Kumar (2017)	Lee (2014)	Willems (2019)	Hernigou (2025)
Type of arthroplasty	Mobile bearing UKA	Mobile bearing UKA	Fixed bearing UKA	Mobile bearing UKA	Mobile bearing UKA	Mobile bearing UKA	TKA	TKA	PFA
Number of knees	98	1000	234	78	1000	369	1500	408	100
Female Pt. (%)	n/a	52	51.45	47.1	52	41	64.6	66	$\approx 51$
Age at surgery	69 (50–79)	66 (32–88)	70 (60–89)	68.8 (48–81) (8.3)	66 (32–88)	69.8 (8.7)	70(34–100)	68.4 (9.5)	65 (45–79)
Follow-up	3.5 (0.33–8)	6.4 (2.9)	10(3.4)	n/a	10.3 (5.3–16.6)	10 (2.9)	4.75 (2–10)	5 (4.75–7)	22(20–25)
Prevalence of CC	20.4	12.6	36 (63)	n/a	13	15.2	26.4	15.4	35

*UKA* Unicompartmental knee arthroplasty, *PFA* patello femoral arthroplasty, *CC* chondrocalcinosis

Among the strategies examined, patellar resurfacing consistently led to a notable decrease in postoperative effusion, whereas synovectomy alone did not prevent moderate-to-severe effusion and was associated with higher complication rates and poorer functional outcomes. These results suggest that, in calcium pyrophosphate deposition disease (CPPD), managing crystal-laden cartilage may be more important than synovial removal in controlling postoperative inflammation.

### Limitations

If an accurate assessment of knee effusion is necessary for diagnosis, obtaining reliable joint fluid samples after total knee arthroplasty can be challenging, and volume determination is difficult with MRI due to artifacts [26]. As a result, the exact volume of knee effusion following TKA remains poorly defined in many cases. This study has other limitations. Its observational design prevents definitive causal conclusions, despite the use of matching and multivariate analysis. Grading of chondrocalcinosis severity, although based on intraoperative and histological criteria, remains semi-quantitative and may cause some observer variability. Postoperative effusion can have multiple causes beyond CPPD, although strict exclusion of infection, hemarthrosis, and metallosis was performed. Finally, synovial regeneration over time could not be measured, limiting the ability to assess the durability of synovectomy. Despite employing propensity-based matching to reduce baseline imbalances, residual confounding from unmeasured variables inherent to the observational design cannot be fully ruled out.

### Pathophysiological interpretation

Postoperative effusion in CPPD-affected knees is believed to result from ongoing intra-articular inflammation caused by residual crystal deposits. Although synovial hypertrophy and inflammation have traditionally been associated with this condition, these findings suggest that patellar cartilage may be a significant and under recognized source of CPPD crystals. Retention of diseased patellar cartilage may allow continuous crystal shedding into the joint after arthroplasty, sustaining synovial irritation even when the implants are properly positioned. Patellar resurfacing removes this cartilage and may therefore reduce the presence of CPPD crystals in the joint. The significant reduction in both the frequency and volume of postoperative effusion seen in resurfaced knees supports this idea.

### Synovectomy: Limited efficacy and added morbidity

Synovectomy has traditionally been used in severe chondrocalcinosis to decrease inflammation. However, the current study shows that synovectomy alone was insufficient to prevent a significant postoperative effusion. Additionally, synovectomy was linked to higher rates of postoperative complications, such as haematoma, infection, stiffness, and patella baja.

Functional outcomes were also poorer in knees that underwent synovectomy, with decreased range of motion and lower Knee Society scores at follow-up. These findings indicate that the additional surgical trauma from extensive synovial resection may outweigh its potential anti-inflammatory benefits in CPPD disease. Importantly, synovectomy did not provide any long-term benefit in implant survivorship.

### Patellar resurfacing in the context of CPPD

The role of patellar resurfacing in TKA remains debated, with previous studies showing mixed results regarding pain, function, and reoperation rates [9, 10]. However, most current research treats TKA as a uniform procedure and does not consider specific conditions like CPPD.

The current study indicates that biological context is important. In cases of severe chondrocalcinosis, patellar resurfacing seems to offer a specific anti-inflammatory benefit by removing crystal-rich cartilage. This benefit was independent of implant alignment, stability, or positioning, as all of these factors were similar between groups. Importantly, patellar resurfacing did not harm implant survival and was linked to lower rates of patellofemoral reoperations.

### Clinical implications

The ability of patellar resurfacing to lessen both the severity and volume of postoperative effusion thus offers a significant clinical advantage for patients with severe CPPD. These findings indicate that routine synovectomy should not be recommended for this population, whereas patellar resurfacing should be considered part of the surgical plan when severe chondrocalcinosis is present.

In patients with chondrocalcinosis, an elevated CRP level is relatively common in the presence of a substantial joint effusion. This increase in CRP may reflect crystal-induced synovial inflammation rather than infection, and therefore should be interpreted with caution when assessing for periprosthetic joint infection. The correlation analysis in the present study revealed that CRP values were positively correlated with total knee effusion volume. This finding suggests that inflammation amplification is accompanied by the production or resorption of joint fluid. TKA may therefore trigger a mild inflammatory response within the synovium, leading to effusion formation, and effusion volume could serve as a surrogate marker of local inflammatory activity in patients with severe chondrocalcinosis.

In this context, the rise in CRP may reflect calcium pyrophosphate crystal–induced synovitis rather than infection. Such crystal-driven inflammation can mimic infectious patterns biologically and clinically, emphasizing the need for cautious interpretation of inflammatory markers and systematic correlation with imaging findings, aspiration results, and crystal analysis before diagnosing periprosthetic joint infection [27, 28].

## Conclusions

In patients with severe chondrocalcinosis undergoing total knee arthroplasty, patellar resurfacing is linked to a significant reduction in postoperative joint effusion, while synovectomy alone does not provide lasting benefit and may increase complications. These findings support the idea that, in CPPD-related knee disease, removing crystal-laden cartilage might be more effective than synovial excision in reducing postoperative inflammation. Surgical decision-making in TKA should therefore consider not only mechanical alignment and implant design but also the underlying biological condition of the joint.

## Data Availability

No datasets were generated or analysed during the current study.

## Declarations

This study was performed in line with the principles of the Declaration of Helsinki. Informed consent was waived due to the retrospective design of the study.
